# Dietary Phenolic Acids and Their Major Food Sources Are Associated with Cognitive Status in Older Italian Adults

**DOI:** 10.3390/antiox10050700

**Published:** 2021-04-29

**Authors:** Justyna Godos, Filippo Caraci, Agnieszka Micek, Sabrina Castellano, Emanuele D’Amico, Nadia Paladino, Raffaele Ferri, Fabio Galvano, Giuseppe Grosso

**Affiliations:** 1 Department of Biomedical and Biotechnological Sciences, University of Catania, 95123 Catania, Italy; justyna.godos@gmail.com (J.G.); fgalvano@unict.it (F.G.); giuseppe.grosso@unict.it (G.G.); 2 Oasi Research Institute-IRCCS, 94018 Troina, Italy; rferri@oasi.en.it; 3 Department of Drug Sciences, University of Catania, 95125 Catania, Italy; 4 Department of Nursing Management and Epidemiology Nursing, Faculty of Health Sciences, Jagiellonian University Medical College, 31-501 Krakow, Poland; agnieszka.micek@uj.edu.pl; 5 Department of Educational Sciences, University of Catania, 95124 Catania, Italy; sabrina.castellano@unict.it; 6 Department of Medical and Surgical Sciences and Advanced Technologies “GF Ingrassia”, University of Catania, 95123 Catania, Italy; emanuele.damico@unict.it; 7 University of Pisa, 56126 Pisa, Italy; nadia.paladino97@gmail.com

**Keywords:** phenolic acids, hydroxycinnamic acids, hydroxybenzoic acids, ferulic acid, caffeic acid, cognitive status, neuroinflammation, coffee, tea, cohort

## Abstract

Background: Life expectancy is increasing along with the rising prevalence of cognitive disorders. Among the factors that may contribute to their prevalence, modifiable risk factors such as diet may be of primary importance. Unarguably, plant-based diets rich in bioactive compounds, such as polyphenols, showed their potential in decreasing risk of neurodegenerative disorders. Therefore, the aim of the present study is to investigate whether exposure to components of plant-based diets, namely phenolic acids, may affect cognitive status in older Italian adults. Methods: The demographic, lifestyle and dietary habits of a sample of individuals living in southern Italy were analyzed. Dietary intake was assessed through food frequency questionnaires (FFQs). Data on the phenolic acids content in foods were estimated using the Phenol-Explorer database. Cognitive status was evaluated using The Short Portable Mental Status Questionnaire. Multivariate logistic regression analyses were used to assess the associations. Results: The mean intake of phenolic acids was 346.6 mg/d. After adjustment for potential confounding factors, individuals in the highest quartile of total phenolic acid intake were less likely to have impaired cognitive status (OR = 0.36 (95% CI: 0.14, 0.92)); similarly, the analysis for subclasses of phenolic acids showed the beneficial effect toward cognitive status of greater intake of hydroxycinnamic acids (OR = 0.35 (95% CI: 0.13, 0.91)). Among individual compounds, only higher intake of caffeic acid was inversely associated with impaired cognitive status (OR = 0.32 (95% CI: 0.11, 0.93)); notably, the association with ferulic acid intake was significant only when adjusting for background characteristics, and not for adherence to the Mediterranean diet. Conclusions: This study revealed that greater intakes of dietary phenolic acids were significantly inversely associated with impaired cognition, emphasizing the possible role of phenolic acids in the prevention of cognitive disorders.

## 1. Introduction

Dietary patterns characterized by a high content in plant-based foods are considered a gold standard to maintain health and reduce the risk of non-communicable diseases [1,2]. The main features of a healthy diet include consumption of fruit and vegetables, nuts, and wholegrains as major food groups exerting positive effects on human health [3,4,5]. In fact, current evidence has underlined the importance of their content in fiber, vitamins and non-vitamin antioxidants in contributing to the health-promoting effects of plant-based dietary patterns [6]. In addition, emphasis has been focused on plant-based beverages, such as tea and coffee, which are consumed worldwide and have demonstrated peculiar benefits for disease prevention [7,8]. A common characteristic of the aforementioned food and beverage groups is the relatively high content in polyphenols. These phytochemicals are widespread in nature and have a number of beneficial effects for plants and as more, were demonstrated to exert positive effects for humans as well. Phytochemicals have been the focus of recent research further supporting the health benefits of plant-based foods and beverages [9].

Observational studies conducted on humans exploring the health benefits of phenolic acids are limited, as research focus mostly on flavonoids and their potential capacity to reduce the risk of hypertension [10], type-2 diabetes [11], and cardiovascular diseases (CVD) [12,13]. Phenolic acids are compounds containing a phenolic ring and an organic carboxylic acid function (C6–C1 skeleton). Phenolic acids are classified as hydroxybenzoic acids (which include individual compounds such as gallic, vanillic, protocatechuic, syringic and salicylic acids) and hydroxycinnamic acids (which include cinnamic, p-coumaric, ferulic, rosmarinic, caffeic and chlorogenic acid). The major dietary sources of phenolic acids include fruits, wholegrains and nuts, cocoa as well as beverages such as coffee and beer [14]. Findings from observational studies on phenolic acid intake have shown an inverse association of their consumption with hypertension [15], type-2 diabetes [16], metabolic syndrome [17], and non-alcoholic fatty liver disease [18]. However, data on outcomes other than cardio-metabolic diseases is quite limited. A plausible effect of total phenolic acids and hydroxycinnamic acids intake was found toward depressive symptoms [19] and sleep quality [20], respectively. Regarding cognitive health, only two studies explored the association between phenolic acid intake and cognitive status; nonetheless, one of the studies provided estimates for total phenolic acid intake, while the other one only for hydroxybenzoic acids. Therefore, the aim of this study was to provide a comprehensive view on the association between dietary phenolic acid intake and cognitive status, considering not only their total intake but also the effect of individual compounds in a cohort of older Italian adults.

## 2. Materials and Methods

### 2.1. Study Population

The MEAL study is an observational study focusing on the link between nutritional and lifestyle habits typical to the Mediterranean area and non-communicable diseases. The study protocol has been described elsewhere [21]. Briefly, the study enrollment took place between 2014 and 2015 in the main districts of the city of Catania in southern Italy. A sample of 2044 men and women aged 18 or more years old was randomly selected and included in the baseline data. Data collection was carried out on using the registered records of local general practitioners stratified by sex and 10-year age groups. In order to provide a specific relative precision of 5% (Type I error, 0.05; Type II error, 0.10), considering an anticipated 70% participation rate, the theoretical sample size was estimated to 1500 individuals.

Out of 2405 individuals invited to participate in the study, 361 refused leaving 2044 participants (response rate of 85%) as the final sample. Taking into consideration that the investigated outcome is more prevalent in the older population, the analysis for the present study was limited to individuals 50 years old or older (*n* = 916). All participants were familiarized about goals of the MEAL study and informed written consent was obtained. The study protocol has been refereed and approved by the concerning ethical committee and all the study procedures were conducted in accordance with the Declaration of Helsinki (1989) of the World Medical Association.

### 2.2. Data Collection

A face-to-face assisted personal interview was conducted during which all participants were provided with a paper copy of the questionnaire in order to visualize the response options. However, the final answers were registered directly by the interviewer using tablet computers. Information regarding sociodemographic and lifestyle factors was collected [22]. The sociodemographic data included age at recruitment, sex, and educational status (low (primary/secondary school), medium (high school), and high (university)). Lifestyle variables included physical activity (low, moderate, high; determined by using the International Physical Activity Questionnaire (IPAQ) [23]), smoking status (non-smoker, ex-smoker, and current smoker), and alcohol drinking (none, moderate drinker (0.1–12 g/d) and regular drinker (>12 g/d)).

### 2.3. Dietary Assessment

Aiming to determine the dietary intake, two food frequency questionnaires (FFQ, a long and a short version) previously proved for validity and reliability for the Sicilian population were adapted [24,25]. Employing the determination of the food intake, the energy content as well as the macro- and micronutrients intake was calculated using food composition tables of the Italian Research Center for Foods and Nutrition [26]. Intake of seasonal foods referred to consumption during the period in which the food was available adjusting by its proportional intake over one year.

Diet quality was evaluated by means of adherence to the Mediterranean diet [27]. Briefly, a literature-based scoring system, which is based on the frequency of intake of different food groups was used. In particular, food groups characteristic to the Mediterranean dietary pattern (such as fruit, vegetables, cereals, legumes, fish, and olive oil) were given positive points, while food groups not representing it (such as meat and dairy products) were given negative points; moderate alcohol intake was deemed as optimal for higher adherence. The final score indicative of adherence to the Mediterranean diet was based on nine food categories with a score ranging from 0 (lowest adherence) to 18 points (highest adherence), and individuals clustered in tertiles categorized as low, medium, and high adherent to the Mediterranean diet [28]. FFQs with unreliable intakes (<1000 or >6000 kcal/d) were excluded from the analyses (n = 33) leaving a total of 883 individuals included in the analysis.

### 2.4. Estimation of Phenolic Acid Consumption

The process of the estimation of dietary phenolic acid intake has been previously described in detail [29]. Briefly, data on the phenolic acid content in plant-based foods and beverages were retrieved from the Phenol-Explorer database [14]. First, the food and beverage consumption was calculated (in g or mL) by following the standard portion sizes used in the study and then converted in 24-h intake. Afterwards, the Phenol-Explorer database was searched to retrieve mean content values for all phenolic acids contained in the applicable food items included in the FFQ, and phenolic acid intake from each food was determined by multiplying the content of total, main subclasses, and selected individual phenolic acids by the daily consumption of each food adjusted for total energy intake (kcal/d) using the residual method [30].

### 2.5. Cognitive Status Evaluation

Cognitive status assessment in the MEAL study was performed using the Short Portable Mental Status Questionnaire (SPMSQ) [31], which was designed to measure cognitive impairment in both the general and hospital population [32], and previously applied in the Italian population [33]. SPMSQ, a 10-item tool, was administered by the clinician in the office or in a hospital. The predefined domains for assessment of the screening tool were the following: (i) intact, less than 3 errors; (ii) mild, 3–4 errors; (iii) moderate, 5–7 errors, and (iv) severe, 8 or more errors. For this study, we considered more than 2 errors as a cut-off point for impaired cognitive status.

### 2.6. Statistical Analysis

Categorical variables are presented as frequencies of occurrence and percentages; differences between groups were tested with Chi-squared test. Continuous variables are presented as means and standard deviations (SDs) or median and standard errors (SEs) whether they were distributed normally or skewed, respectively; differences between groups were tested with Student’s *t*-test or Mann–Whitney U-test for variables distributed normally or skewed, respectively. The relation between exposure variables and cognitive status was tested through uni- and multivariate logistic regression analysis adjusted for baseline characteristics (age, sex, educational status, smoking and alcohol drinking habits, and physical activity level). An additional model was performed to further adjust for adherence to the Mediterranean diet, as a proxy variable for diet quality. The association between major dietary sources of phenolic acids and cognitive status was finally evaluated. All reported *p*-values were based on two-sided tests and compared to a significance level of 5%. SPSS 17 (SPSS Inc., Chicago, IL, USA) software was used for all the statistical calculations.

## 3. Results

**Figure 1:**
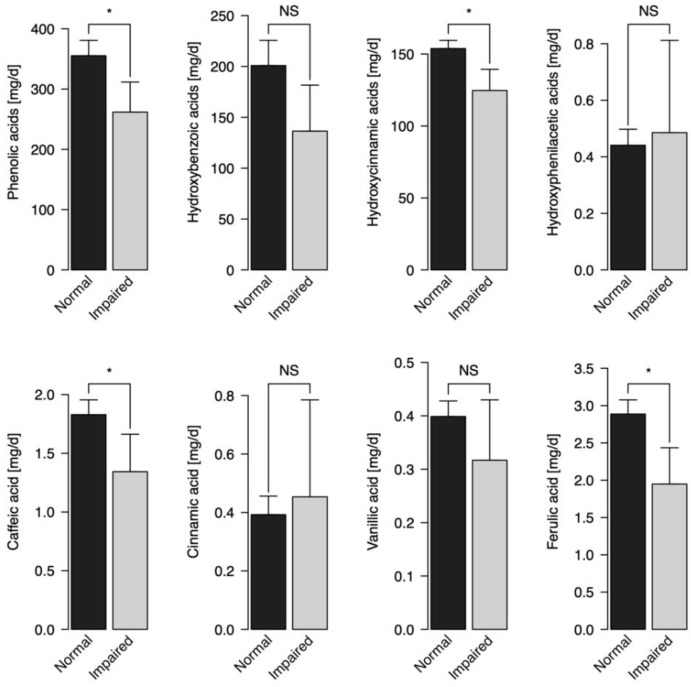
Comparison of the mean intake of total, subgroups, and individual phenolic acids by cognitive status. Barplots represent mean values of phenolic acids intake, while whiskers represent upper bounds of 95% confidence intervals for mean values of phenolic acids intake. NS—not significant; * *p* < 0.05.

A total of 883 individuals were included in the analyses of this study. The mean age was 64.9 years old, 56.7% were female. Eighty individuals (9.1% of the sample) were categorized as having impaired cognitive status. The mean intake of phenolic acids was 346.6 mg/d. Comparison of the mean intake of total, subgroups, and individual phenolic acids by cognitive status is reported in Figure 1.

The mean intake of all the types of phenolic acids was significantly higher in individuals with normal cognitive status when comparing with individuals with impaired cognitive status (Figure 1): total phenolic acids (355.3 mg/d (95% CI: 329.9, 380.69) vs. 261.8 mg/d (95% CI: 212.04, 311.57)), hydroxycinnamic acids (153.77 mg/d (95% CI: 148.1, 159.43) vs. 124.62 mg/d (95% CI: 109.9, 139.34)), caffeic acid (1.83 mg/d (95% CI: 1.7–1.95) vs. 1.34 mg/d (95% CI:1.02, 1.66)) and ferulic acid (2.89 mg/d (95% CI: 2.7, 3.08) vs. 1.95 mg/d (95% CI: 1.46, 2.44)).

To test whether intake of phenolic acid was distributed differently across potential confounding factors, the main background characteristics of the study sample categorized by quartiles of phenolic acid intake are presented in Table 1. The age of study participants varied when considering level of the exposure to dietary phenolic acid intake. There were significant differences in the distribution of the variables such as smoking status and physical activity level among the quartiles of phenolic acids intake, however with no particular trends toward healthier or unhealthier behaviors. Additionally, there was a higher proportion of regular alcohol drinkers among the highest quartile of phenolic acid intake. When considering the quartiles with greater phenolic acid intake, a higher proportion of individuals with high adherence to the Mediterranean diet was observed (Table 1).

After adjustment for potential confounding factors, individuals in the highest quartile of total phenolic acid intake (median intake = 509.2 mg/day) were less likely to have impaired cognitive status than those consuming the least (OR = 0.36 (95% CI: 0.14, 0.92)), although there was no clear trend across quartiles of exposure (Table 2). The analysis for subclasses of phenolic acids showed that individuals with the highest intake of hydroxycinnamic acids (highest vs. lowest category, OR = 0.35 (95% CI: 0.13, 0.91)) were less likely to have impaired cognitive status (Table 2). Moreover, a similar inverse association was also found for the third versus the first quartile of intake of hydroxyphenylacetic acid (OR = 0.26 (95% CI: 0.11, 0.63)). Among individual compounds investigated, only higher intake of caffeic acid resulted inversely associated with impaired cognitive status (OR = 0.32 (95% CI: 0.11, 0.93)); notably, the association with ferulic acid intake was significant when adjusting for background characteristics, but further adjustment for adherence to the Mediterranean diet nullified the results (OR = 0.42 (95% CI: 0.16, 1.07)).

The association between major dietary sources of phenolic acids and cognitive status is shown in Table 3. Individuals in the highest category of consumption of coffee (OR = 0.44 (95% CI: 0.20, 0.98)) and tea (OR = 0.30 (95% CI: 0.12, 0.72)) were less likely to have impaired cognitive status. On the contrary, no significant association was found for olive oil, nuts and beer intake.

## 4. Discussion

To our knowledge, this is the first observational study to examine the association between total dietary phenolic acids and their individual compounds intake and cognitive status. We observed that higher intakes of total phenolic acids and hydroxycinnamic acids were significantly inversely associated with cognitive impairment. Furthermore, among individual compounds, caffeic acid and ferulic acid showed a potential beneficial effect toward cognitive status.

Up to date, solely two observational studies investigated the association between dietary phenolic acid intake and cognitive status [34,35]. A prospective cohort study of 806 elderlies followed-up for 6 years showed a significant inverse association between higher dietary phenolic acid intake and cognitive decline, however, a clear linear trend in the association was not observed [34]. The results of SU.VI.MAX study conducted on 2574 middle-aged adults demonstrated that higher intake of hydroxybenzoic acids was positively associated with language and verbal memory, suggesting that diet high in phenolic acids may favor preservation of the verbal memory [35].

In accordance with the literature, the results of this study showed that intake of beverages containing phenolic acids, such as coffee and tea, may significantly impact cognitive status [36,37]. However, when interpreting these results it should be taken into consideration that the average daily intake of phenolic acids and their major food sources greatly differs across European populations [29,38,39]: in fact, to our knowledge, the present study is the first reporting such results in a population with very low consumption of tea, and relatively low consumption of coffee, compared to northern European countries, thus these results should be interpreted in this context.

Consistent with evidence from experimental studies, the results found in the present study suggest that individuals with high intake of hydroxycinnamic acids, as well as individual compounds such as caffeic acid and ferulic acid are less likely to have impaired cognitive status. From a mechanistic point of view, phenolic acids may affect cognitive function through various pathways including both direct and indirect modes of action. Phenolic acids have been shown to modulate different factors associated with the pathogenesis of neurodegenerative diseases, such as systemic inflammation and oxidative stress, and thus indirectly affect cognitive health [40]. As suggested by the evidence from experimental studies, specific phenolic acids and their metabolites (i.e., caffeic acid, ferulic acid) have been found in cerebrospinal fluid, suggesting that these compounds may cross the blood–brain barrier and thus directly affect brain cells [41,42]. Moreover, new evidence suggests that there is an interaction between dietary polyphenols and gut microbiota and their effect toward the brain [43,44]: it has been determined that gut microbiota composition significantly affects the bioavailability of polyphenols including phenolic acids and consequently may mediate and modulate the effect of dietary polyphenols toward cognitive health [45,46].

Concerning the direct effects of phenolic acids on the central nervous system, it has been demonstrated that hydroxycinnamic acids and their esters may exert beneficial effects toward cognitive functions by improving neuronal cell antioxidant activity against oxidative stress [47,48], through various signaling pathways including Nrf2/HO-1 pathway [49]. Moreover, hydroxycinnamic acids have been shown to rescue memory deficits in animal models of diabetes induced by streptozotocin through the reduction of oxidative stress and the positive modulation of the PI3-kinase pathway [50,51]. Among hydroxybenzoic acids, gallic acid has been shown to reverse impaired learning and memory in a rodent model of Alzheimer’s disease through simultaneous elevation of α-secretase and reduction of β-secretase activity and, most importantly, activation of metalloproteinase domain-containing protein 10 [52]. Furthermore, its role in modulation of neuroinflammation and the prevention of brain oxidative stress has been suggested. Recent studies also suggest that gallic acid can bind beta-amyloid monomers and prevent beta-amyloid aggregation, a primary event in the pathophysiology of Alzheimer’s disease [53]. Similarly, an increase in hippocampal dendritic spines and decreased oxidative stress and inflammation has been observed after administration of gallic acid in rats with metabolic syndrome [54], indicating that gallic acid could also play a protective role in the prevention of cognitive impairment triggered, at least partially, by metabolic disorders. Ellagic acid, which belongs to hydroxybenzoic acids, has been shown to exert neuroprotective effects by modulating neuroinflammation is a promising constituent for improving cognitive impairment [55] and alleviating memory impairment [56]. The neuroprotective activity of ellagic acid has been also validated in APP/PS1 mice, a validated transgenic animal model of Alzheimer’s disease, where it prevents neuronal apoptosis in the hippocampus and rescues memory deficits through the inhibition of beta-amyloid production and tau hyperphosphorylation [57].

Ferulic acid has been shown to promote neural differentiation and neurite outgrowth by exerting antiapoptotic and neuronal differentiation-inducing effects in a dose-dependent manner [58], while caffeic acid esters were found to influence the expression of brain-derived neurotrophic factor, a neurotrophic factor implicated in neuron survival, adult neurogenesis and synaptic plasticity [59]. The neuroprotective activity of ferulic acid is well-established and it is currently adopted as a potential pharmacophore in drug discovery processes for Alzheimer’s disease due to its multimodal mechanism of action which combines antioxidant and antiaggregant effects against Aβ oligomers with a Cholinesterase inhibition and a significant anti-inflammatory activity [60]. Different ferulic acid-based hybrid analogs are currently studied as multitarget directed ligands to develop novel hybrid compounds against Alzheimer’s disease [60].

Notwithstanding the novelty of this study, which for the first time explored the association between total phenolic acid and their individual components intake and cognitive status among the adult population, the results should be considered in light of some limitations.

First, given the cross-sectional design of the study, we cannot define the causal relation. Second, despite adjustment for potential confounders, including dietary factors, even though unlikely, residual confounding may be possible. However, after adjustment for the adherence to the Mediterranean diet most of the associations remained significant. Third, the use of FFQs may be susceptible to recall bias and potential inaccuracy in estimating dietary intake at least partially attributable to interpersonal variability in phenolic acids intake directly related to food quality (i.e., plant variety, season and environmental factors, food storage and processing). Finally, interindividual differences in the bioavailability and physiological response to phenolic acids exposure cannot be established [61].

## 5. Conclusions

In the present Mediterranean cohort, greater intakes of dietary phenolic acids were significantly inversely associated with impaired cognition. This association was confirmed for different phenolic acid subclasses and several individual compounds, emphasizing the importance of investigating a broad range of compounds in future studies to better elucidate their effect toward cognitive function. If confirmed, these findings may have important implications for cognitive decline prevention, given that the availability of modifiable risk factors which may impact cognition is limited.

## Figures and Tables

**Table 1:** Background characteristics by quartiles of dietary phenolic acid intake in the study sample (*n* = 883).

	Phenolic Acid Intake				
	Q1, *n* = 219 (Mean = 114.4 mg/d)	Q2, *n* = 220 (Mean = 202.7 mg/d)	Q3, *n* = 208 (Mean = 306.6 mg/d)	Q4, *n* = 236 (Mean = 711.8 mg/d)	*p*-Value
Age groups, mean (SD)	67.1 (10.3)	65.6 (9.8)	63.1 (8.2)	64.1 (9.5)	<0.001
Sex, *n* (%)					0.053
Men	46 (33.6)	67 (46.2)	49 (33.8)	63 (43.4)	
Women	91 (66.4)	78 (53.8)	96 (66.2)	82 (56.6)	
Educational status, *n* (%)					0.812
Low	88 (64.2)	86 (59.3)	80 (55.2)	88 (60.7)	
Medium	29 (21.2)	38 (26.2)	41 (28.3)	34 (23.4)	
High	20 (14.6)	21 (14.5)	24 (16.6)	23 (15.9)	
Smoking status, *n* (%)					0.001
Never smoker	84 (61.3)	76 (52.4)	93 (64.1)	87 (60.0)	
Former smoker	15 (10.9)	39 (26.9)	35 (24.1)	29 (15.2)	
Current smoker	38 (27.7)	30 (20.7)	17 (11.7)	36 (24.8)	
Physical activity level, *n* (%)					<0.001
Low	45 (41.3)	39 (32.0)	41 (33.9)	21 (18.8)	
Moderate	34 (31.2)	63 (51.6)	71 (58.7)	70 (62.5)	
High	30 (27.5)	20 (16.4)	9 (7.4)	21 (18.8)	
Alcohol intake, *n* (%)					<0.001
No	28 (20.4)	46 (31.7)	40 (27.6)	25 (17.2)	
Moderate	96 (70.1)	78 (53.8)	66 (45.5)	61 (42.1)	
Regular	13 (9.5)	21 (14.5)	39 (26.9)	59 (40.7)	
Mediterranean diet adherence, *n* (%)					<0.001
Low	54 (27.7)	55 (24.6)	26 (11.5)	33 (13.9)	
Medium	64 (32.8)	63 (28.1)	65 (28.6)	65 (27.4)	
High	77 (39.5)	106 (47.3)	136 (59.9)	139 (58.6)	

**Table 2:** Association between total, main classes, and individual phenolic acid intake and impaired cognitive status.

	Phenolic Acid Intake			
	Q1	Q2	Q3	Q4
Phenolic acids, median (SE), mg/day	122.1 (2.4)	205.2 (1.8)	302.9 (2.5)	509.2 (34.0)
Model 1, OR (95% CI) ^a^	1	0.33 (0.17–0.64)	0.51 (0.28–0.94)	0.34 (0.17–0.71)
Model 2, OR (95% CI) ^b^	1	0.26 (0.12–0.57)	0.69 (0.32–1.45)	0.35 (0.14–0.89)
Model 3, OR (95% CI) ^c^	1	0.27 (0.13–0.58)	0.74 (0.34–1.57)	0.36 (0.14–0.92)
Hydroxybenzoic acids, median (SE), mg/day	8.8 (0.8)	64.0 (0.5)	131.6 (2.1)	284.2 (34.4)
Model 1, OR (95% CI) ^a^	1	0.83 (0.45–1.53)	0.77 (0.40–1.48)	0.73 (0.38–1.41)
Model 2, OR (95% CI) ^b^	1	0.84 (0.40–1.74)	0.82 (0.38–1.77)	1.01 (0.45–2.22)
Model 3, OR (95% CI) ^c^	1	0.84 (0.40–1.75)	0.82 (0.38–1.78)	1.00 (0.45–2.22)
Hydroxycinammic acids, median (SE), mg/day	66.3 (1.3)	105.9 (0.9)	154.4 (1.1)	243.0 (4.8)
Model 1, OR (95% CI) ^a^	1	1.00 (0.57–1.77)	0.35 (1.78–0.71)	0.43 (0.20–0.92)
Model 2, OR (95% CI) ^b^	1	0.83 (0.43–1.61)	0.26 (0.11–0.59)	0.35 (0.14–0.88)
Model 3, OR (95% CI) ^c^	1	0.83 (0.43–1.62)	0.26 (0.11–0.62)	0.35 (0.13–0.91)
Hydroxyphenylacetic acids, median (SE), mg/day	0.0 (0.0)	0.2 (0.0)	0.4 (0.004)	0.8 (0.1)
Model 1, OR (95% CI) ^a^	1	0.93 (0.51–1.68)	0.42 (0.20–0.88)	0.82 (0.43–1.58)
Model 2, OR (95% CI) ^b^	1	0.47 (0.23–0.97)	0.26 (0.11–0.61)	0.45 (0.18–1.10)
Model 3, OR (95% CI) ^c^	1	0.48 (0.24–0.99)	0.26 (0.11–0.63)	0.46 (0.19–1.11)
Caffeic acid, median (SE), mg/day	0.4 (0.0)	0.8 (0.0)	1.4 (0.0)	3.3 (0.1)
Model 1, OR (95% CI) ^a^	1	0.51 (0.28–0.93)	0.26 (0.13–0.53)	0.46 (0.24–0.89)
Model 2, OR (95% CI) ^b^	1	0.57 (0.27–1.18)	0.28 (0.12–0.63)	0.31 (0.11–0.92)
Model 3, OR (95% CI) ^c^	1	0.58 (0.28–1.21)	0.29 (0.12–0.68)	0.32 (0.11–0.93)
Cinnamic acid, median (SE), mg/day	0.0 (0.0)	0.1 (0.0)	0.2 (0.0)	0.5 (0.1)
Model 1, OR (95% CI) ^a^	1	0.82 (0.43–1.54)	0.98 (0.52–1.84)	0.74 (0.38–1.44)
Model 2, OR (95% CI) ^b^	1	0.63 (0.29–1.36)	1.02 (0.50–2.08)	0.57 (0.27–1.22)
Model 3, OR (95% CI) ^c^	1	0.65 (0.30–1.39)	1.04 (0.51–2.11)	0.60 (0.28–1.29)
Vanillic acid, median (SE), mg/day	0.1 (0.0)	0.1 (0.0)	0.4 (0.0)	0.8 (0.0)
Model 1, OR (95% CI) ^a^	1	0.50 (0.26–0.96)	0.51 (0.27–0.96)	0.59 (0.31–1.14)
Model 2, OR (95% CI) ^b^	1	0.34 (0.15–0.74)	0.46 (0.21–1.00)	0.43 (0.17–1.05)
Model 3, OR (95% CI) ^c^	1	0.34 (0.15–0.74)	0.48 (0.22–1.06)	0.45 (0.18–1.11)
Ferulic acid, median (SE), mg/day	0.6 (0.0)	1.4 (0.0)	2.7 (0.0)	5.5 (0.2)
Model 1, OR (95% CI) ^a^	1	0.65 (0.35–1.20)	0.75 (0.42–1.35)	0.32 (0.14–0.73)
Model 2, OR (95% CI) ^b^	1	0.73 (0.36–1.47)	1.04 (0.50–2.15	0.39 (015–0.98)
Model 3, OR (95% CI) ^c^	1	0.76 (0.37–1.55)	1.13 (0.53–2.39)	0.42 (0.16–1.07)

^a^ Model 1 adjusted for energy intake (kcal/day, continuous); ^b^ Model 2 = Model 1 + adjusted for BMI (continuous), smoking status (smokers, ex-smokers, non-smokers), alcohol consumption (0 g/day, <12 g/day, ≥12 g/day), physical activity level (low, medium, high), educational level (low, medium, high); ^c^ Model 3 = Model 2 + adherence to the Mediterranean diet. OR (odds ratio); CI (confidence interval).

**Table 3:** Association between major dietary food sources of phenolic acids and impaired cognitive status.

	Food Group Intake, OR (95% CI) ^a^			
	Q1	Q2	Q3	Q4
Coffee ^b^	1	0.52 (0.12–2.14)	0.96 (0.45–2.04)	0.44 (0.20–0.98)
Nuts ^c^	1	1.52 (0.71–3.27)	1.19 (0.54–2.62)	1.16 (0.48–2.77)
Tea ^d^	1	0.76 (0.38–1.53)	0.39 (0.18–0.82)	0.30 (0.12–0.72)
Olive oil ^e^	1	1.01 (0.43–2.35)	1.37 (0.60–3.08)	-
Beer ^f^	1	0.81 (0.42–1.57)	1.22 (0.43–3.47)	1.63 (0.43–6.15)

^a^ OR adjusted for sex, age (years, continuous), energy intake (kcal/day, continuous), smoking status (smokers, ex-smokers, non-smokers), alcohol consumption (0 g/day, <12 g/day, ≥12 g/day), physical activity level (low, medium, high), educational level (low, medium, high); ^b^ categories of coffee intake were as follows: Q1, less than 3 cups/week; Q2, 3 cups/week to 1 cup/day; Q3, more than 1 to less than 3 cups/day Q4, more than 3 cups/day; ^c^ categories of nuts intake were as follows: Q1, none; Q2, less than 1 serving (28 g)/day; Q3, 1–2 servings/day; Q4, more than 2 servings/day; ^d^ categories of tea intake were as follows: Q1, none; Q2, less than 1 cup/week; Q3, more than 1 cup/week to 1 cup/day; Q4, more than 1 cup/day; ^e^ categories of olive oil intake were as follows: Q1, less than 1 serving (1 spoon)/day; Q2, ½ to 1 serving/day; Q3, more than 1 serving/day; ^f^ categories of beer intake were as follows: Q1, none; Q2, less than 1 can/week; Q3, 1–3 cans/week; Q4, more than 3 cans/week.

## Data Availability

Data is contained within the article.
